# Risk assessment based on indirect predation cues: revisiting fine‐grained variation

**DOI:** 10.1002/ece3.1552

**Published:** 2015-09-27

**Authors:** Michael W. McCoy, Stefan K. Wheat, Karen M. Warkentin, James R. Vonesh

**Affiliations:** ^1^ Department of BiologyVirginia Commonwealth UniversityRichmondVA; ^2^ Department of BiologyEast Carolina UniversityGreenvilleNC; ^3^ Department of BiologyWhitman CollegeWalla WallaWA; ^4^ Department of BiologyBoston UniversityBostonMA; ^5^ Smithsonian Tropical Research InstituteGamboaPanama

**Keywords:** Inducible defense, phenotypic plasticity, predation, tadpole

## Abstract

To adaptively express inducible defenses, prey must gauge risk based on indirect cues of predation. However, the information contained in indirect cues that enable prey to fine‐tune their phenotypes to variation in risk is still unclear. In aquatic systems, research has focused on cue concentration as the key variable driving threat‐sensitive responses to risk. However, while risk is measured as individuals killed per time, cue concentration may vary with either the number or biomass killed. Alternatively, fine‐grained variation in cue, that is, frequency of cue pulses irrespective of concentration, may provide a more reliable signal of risk. Here, we present results from laboratory experiments that examine the relationship between red‐eyed treefrog tadpole growth and total cue, cue per pulse, and cue pulse frequency. We also reanalyze an earlier study that examined the effect of fine‐grained variation in predator cues on wood frog tadpole growth. Both studies show growth declines with increasing cue pulse frequency, even though individual pulses in high‐frequency treatments contained very little cue. This result suggests that counter to earlier conclusions, tadpoles are using fine‐grained variation in cue arising from the number of predation events to assess and respond to predation risk, as predicted by consumer–resource theory.

## Introduction

Many species modify their phenotype in response to cues that indicate predation risk, including indirect visual, auditory, or chemical cues that arise from predation events or attempts (Chivers and Smith 1998; Tollrian and Harvell 1999). This ability to tune phenotypes can enhance fitness when predation risk is variable and there are trade‐offs for expressing defended phenotypes when risk is low (Riessen 1992). To respond adaptively, prey must be able to accurately gauge and adjust to variation in risk based on information provided by indirect cues (Chivers and Smith 1998; Luttbeg and Trussell 2013). There is considerable evidence that prey are able to do just that. Prey modulate responses according to predator identity (Relyea 2001; Vonesh and Warkentin 2006; Touchon and Warkentin 2008), diet (Schoeppner and Relyea 2005, 2009), conspecific density (McCoy 2007; Van Buskirk et al. 2011), and number (McCoy et al. 2012) and biomass of prey consumed (Fraker 2008; McCoy et al. 2012). However, the information contained in indirect cues that enable prey to assess variation in risk is still unclear (Fraker 2008).

Much of the empirical work on predator‐induced defenses has considered only cue presence or absence, often crossed with different environmental contexts (e.g., varying resources or conspecific densities). A smaller number of studies have examined graded response to variation in cue magnitude. For example, in aquatic systems studies have manipulated cue concentration by varying the biomass of prey consumed by predators per unit time, often showing prey responses increase with increasing cue (e.g., Fraker 2008; Tollrian 1993). However, linking cue concentration to risk is problematic, as biomass consumed can increase as either a function of the number of prey or the size of prey (McCoy et al. 2012). Most consumer–resource theory predicts per capita risk of predation to be a function of number of prey consumed per time. Indeed, in a recent study we showed that for a given total biomass of prey consumed, prey responded more strongly to predators consuming multiple smaller prey than a single larger prey (McCoy et al. 2012). This result suggests that fine‐scale variation in frequency of predation events provides additional information about risk beyond the amount of cue. Thus, while cue concentration provides information about either the size or the number of prey consumed, predation frequency provides information on predation events irrespective of prey size. If predation frequency provides less ambiguous information about risk, we might expect prey to respond more strongly to this aspect of chemical cues.

**Figure 1:**
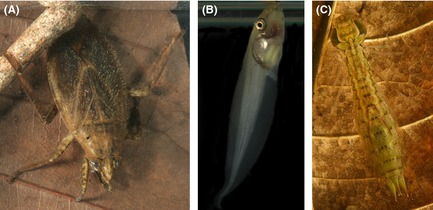
Study organisms. (A) Giant water bug (*Belostoma cf porteri*), (B) Red‐eyed treefrog (*Agalychnis callidryas*) tadpole, and (C) Amazon darner (*Anax amazili*) dragonfly nymph.

Here, we conducted a pair of laboratory experiments with red‐eyed treefrog tadpoles and two common predators (Fig. 1), to disentangle the relationships between cue concentration, fine‐grained variation in cue pulse frequency, and prey growth response. First, we test whether prey response increases with cue concentration, controlling for fine‐grained variation (i.e., the number of cue pulses). We then test the effects of cue pulse frequency and cue concentration per pulse, controlling for the total cue delivered. We also re‐examine results from a similar experiment conducted with wood frogs (Schoeppner and Relyea 2009). If prey responses are primarily based on information provided by cue concentration, we expect an increasingly negative effect of cue addition with increasing total and individual pulse cue concentration. In contrast, if prey responses are primarily based on the number rather than magnitude of predation events, we would expect prey responses to increase with pulse frequency and, perhaps counter intuitively, to decrease with fewer more concentrated pulses.

## Methods

Experiments were conducted in a covered open‐air field laboratory at the Smithsonian Tropical Research Institute (STRI), in Gamboa, Panama. Predators were field‐collected and maintained in the laboratory during experiments. Red‐eyed treefrog tadpoles were field‐collected as eggs, maintained in the laboratory, and induced to hatch 6 days postoviposition (dpo; (Warkentin 2000)). Experiments were conducted with tadpoles in individual containers of 400 mL aged tap water randomly arranged on a laboratory bench. Cue was generated by feeding a predator a 6–8 dpo tadpole (mean total length: 13.8 mm, 0.021 g) in 500 ml aged tap water. Predators were allowed to feed for 30 min after prey capture to release cues into the water; then we pooled cue water from three individual predators to yield 1.5 L of cue at a concentration of 2 tadpoles consumed L^−1^. We diluted this stock of cued water as appropriate to yield the concentration required for each treatment. Water volume was maintained at 400 mL over both experiments. Initial and final tadpole total lengths (TL, mm) were measured from digital photographs using the program ImageJ (NIH). Tadpole length was converted to mass using published relationships (Asquith and Vonesh 2012) and analyses focused on log final mass. Statistical analyses were conducted using R version 2.15.1 (2012‐06‐22) (R Development Core Team 2013).

To test the effects of predator identity and cue concentration on growth, we crossed the identity of the cue‐generating predator (late instar dragonfly larvae – *Anax amazili*; giant water bug – *Belostoma* cf *porteri* Fig. 1) with four total cue levels (0, 0.04375, 0.4375, 0.875 g tadpoles consumed L^−1^ day^−7^) and quantified tadpole growth over 7 days (eight treatments replicated 12 times). Cue was added daily from stock solution prepared 30 min prior to addition. Focal tadpoles came from twelve 2 dpo clutches collected on July 2, 2011. We fed each tadpole 0.05 g Sera micron (Sera, Germany) every other day starting at hatching. We used stepwise linear models to test for the effects of predator identity, total cue concentration, and their interaction on log tadpole final mass.

To test for cue pulse frequency effects on growth, we exposed tadpoles to the same total cue (0.105 g prey consumed L^−1^ day^−7^) delivered at five frequencies and quantified growth after 7 days: (1) no cue, (2) 1 cue pulse of 0.105 tadpoles consumed L^−1^ on day 1, (3) 3 cue pulses of 0.035 added on days 1, 4, and 7, (4) 7 cue pulses of 0.015 added daily, and (5) 21 cue pulses of 0.005 added three times daily (five treatments replicated 12 times). Cue or water control was added every 8 h (0800, 1600, and 2200 h). For treatments that received cue less than three times daily, actual cue was administered at 0800 h. Given the lack of predator‐specific responses above, we focused on water bugs. Tadpoles came from six 1 dpo clutches collected on July 27, 2011. We fed each tadpole 0.02 g Sera micron daily to reduce water fouling. We used linear models to test for the effects of cue pulse frequency on log tadpole final mass.

Schoeppner and Relyea (2009) conducted a similar experiment in 800 L mesocosms in which they quantified wood frog (*Lithobates sylvaticus*) tadpole traits in response to fine‐grained variation in cues from predacious diving beetles while holding total cue delivered over the experiment constant. Tadpoles were exposed to a no‐predator control or an average of 0.004 g L^−1^day^−7^ with predators being fed daily or at 2‐, 4‐, and 8‐day intervals over the 24‐day experiment. We digitally extracted treatment means and ± 1 SE for tadpole final mass (mg) for all nine treatments from Figure 3 (Schoeppner and Relyea 2009). Using ANOVA, the earlier investigation concluded that the fed‐daily predator treatment was not different from any of the more variable predator cue categories. Here, we take a slightly different approach, paralleling our experiments, to examine the relationship between log tadpole final mass and cue pulse frequency (an ordinal ranging from 0 to 24) and mean pulse concentration via regression, rather than testing for significant differences between unordered categories.

## Results

**Figure 2:**
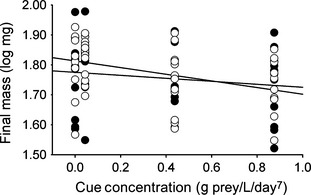
Tadpole final mass as a function of cue concentration from dragonfly (open circles) and water bug (filled circles) predators.

Initial tadpole length ([$\overset{¯}{x}$ ± 1 SD] 11.75 mm ± 0.75, *F*_7,88_ = 0.11, *P* = 0.99) and estimated mass (12.0 mg ±2.0, *F*_7,88_ = 0.15, *P* = 0.99) were not different among treatments in the cue concentration experiment. Nine hatchlings died over the course of the experiment (9%). This mortality was not related to treatment (binomial glm LR *χ* ^2^ = 10.85, df = 7, *P* = 0.15). Across treatments, tadpoles increased total length by 76% (8.94 ± 1.42 mm) and increased mass fivefold (48.63 ± 12.16 mg). The effect of cue concentration on final mass became increasingly negative as total cue addition increased (Total cue coefficient [$\overset{¯}{x}$  ± 1 SD] = −0.186 ± 0.069, *F*_1,85_ = 7.14, *P* = 0.009, Adj *R* ^2^ = 0.07, Fig. 2). Terms for predator identity and the cue concentration by predator identity interaction were not retained.

**Figure 3:**
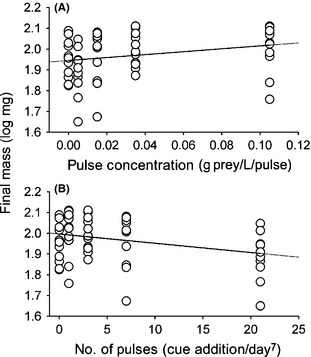
Tadpole final mass as a function of (A) Concentration of individual cue pulses from a water bug predator and (B) Number of pulses over 7 days, holding total cue delivered constant.

Initial tadpole length (11.54 mm ± 0.70, *F*_4,55_ = 0.31, *P* = 0.86) and estimated mass (11.0 mg ± 2.0, *F*_4,55_ = 0.19, *P* = 0.94) were not different among treatments in the cue frequency experiment. All tadpoles survived to the end of the experiment. Across treatments, tadpoles increased total length by 110% (12.69 mm ±1.87) and increased mass nearly ninefold (84.9 mg ± 21.0). While all treatments received the same total amount of cue, the frequency with which cue was delivered was important. The effect of cue on final mass became increasingly negative as the frequency of cue addition increased (Cue frequency coefficient =−0.001 ± 0.0041, *F*_1,58_ = 6.34, *P* = 0.014, *R* ^2^ = 0.084, Fig. 3B). More frequent pulses were of necessarily lower concentration and corresponded with the effect of cue on growth which became increasingly *positive* (not negative) as the concentration of each cue pulse increased (Pulse concentration coefficient = 1.61 ± 0.83, *F*_1,58_ = 3.78, *P* = 0.056, *R* ^2^ = 0.05, Fig. 3A).

**Figure 4:**
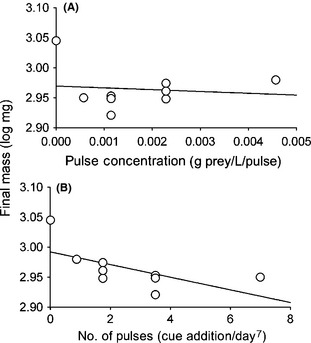
Wood frog final mass as a function of (A). The concentration of each cue pulse and (B) The number of cue pulses over the 7 days. Recreated from Schoeppner and Relyea (2009) Fig. 3A using the combined data extracted from no‐predator controls and treatments with cue addition daily and at 2‐, 4‐, and 8‐day intervals.

The data in Schoeppner and Relyea (2009) are aggregated means for tadpole final mass in each cue pulse treatment. While the sample size is small, the reanalysis of treatment means from Schoeppner and Relyea (2009) nevertheless revealed a qualitatively similar pattern as observed in this study. Final mass of wood frogs tended to decline with increasing cue pulse frequency (Pulse frequency coefficient = −0.0105 ± 0.0049, *F*_1,7_ = 4.46, *P* = 0.073, Adj *R* ^2^ = 0.30, Fig. 4B) and showed no relationship between pulse cue concentration and growth (Pulse concentration coefficient = −3.012 ± 9.71, *F*_1,7_ = 0.096*P* = 0.765, Adj *R* ^2^ = 0.01, Fig. 4A). In treatments where cue was present (i.e., excluding controls), growth increased (not decreased) with increasing cue concentration (Pulse concentration coefficient = 10.13 ± 4.19, *F*_1,6_ = 5.86, *P* = 0.05, Adj *R* ^2^ = 0.41).

## Discussion

Understanding how indirect chemical cues of predation events are translated into useable information about predation risk is key to integrating predator‐induced defenses into consumer–resource theory and for predicting how induced defenses impact population and community dynamics. Consumer–resource theory typically predicts per capita risk of predation to be a function of the number of prey consumed by predators, but most empirical work, particularly in aquatic systems, has assumed that risk assessment is based on cue concentration (e.g., prey biomass consumed time^−1^ vol^−1^). Cue concentrations, however, may be independent of the number of prey consumed (McCoy et al. 2012). In this study, we found that increasing cue pulse frequency reduced growth, even though individual pulses in high‐frequency treatments contained very little cue, suggesting that cue pulse frequency may be more informative than cue concentration for prey risk assessment. This result suggests that tadpoles may assess and respond to predation risk based on the number of predation events as predicted by consumer–resource theory.

There two ways by which tadpoles may be assessing predation risk as a function of the frequency of predation events in their environment. First, pulse frequency may provide less ambiguous information about risk, because as the frequency of predation events increases variability of an indirect cue that decays through time also becomes less variable (Peacor 2003). Alternatively, individuals may be learning from and updating their assessment of risk based on their prior experiences. This can be described quantitatively using a Bayesian learning model (Rodríguez‐Gironés and Vasquez 1997; Olsson and Holmgren 1998; Biernaskie et al. 2009). For example, if we assume predation events follow a Poisson distribution (a reasonable assumption for most predator functional responses) with a mean equal to the average predation rate, *P,* then the posterior probability of predation will follow a Gamma distribution with a mean = P and with a shape parameter equal to the abundance of conspecific prey in the environment. The shape of this distribution provides a description of uncertainty about the predation environment. So, when prey density is high relative to predation events, a high degree of uncertainty about the predation environment would exist and so predation risk would be perceived to be low. However as prey density gets smaller (as prey are consumed), so does the amount of uncertainty in the risk of being depredated (i.e., as the shape parameter of the Gamma distribution approaches zero, the distribution becomes more peaked and thus true predation threat more certain).

Although our findings are consistent with the idea that prey assess risk in part based upon the frequency of predation events, an alternative hypothesis is that tadpoles exposed to fewer cue pulses have more time to compensate for lost foraging time or recover from the negative effects of increased stress hormone levels. Behavioral responses to predator cues are often quite short, fading as cue breaks down or disperses over time (Relyea 2003; Fraker 2008). As the strength of the prey behavioral response fades, a single pulse of cue may lead to a smaller effect on foraging compared with multiple cue pulses even if the cumulative cue strength is the same across frequency treatments and if experiment is of long enough duration to compensate for lost foraging effort (Relyea 2003). Alternatively, even in the absence of reduced foraging activity, repeated exposure to stressors can lead to reduced growth associated with elevated stress hormone levels (McCormick et al. 1998; Denver 2009; Bliley and Woodley 2012). Again, in treatments with fewer predation events, there may be more time for prey to recover. Unfortunately, we lack data on foraging activity changes through the experiment. Tadpoles were filter‐feeding on suspended Sera micron, and we were not able to visually discriminate buccal pumping for respiration and filter– feeding. Nor do we have data on stress hormone levels. Thus, we cannot discount that these mechanisms may also contribute to our results.

Regardless of whether tadpoles are using variation in the cue environment or are learning from past experiences, our results suggest that tadpoles are assessing risk based on the number of predation events occurring in the detectable environment and that the frequency of predation events is an important factor in determining the nonlethal effects of predators on prey growth independent of the magnitude of the cue signal. These findings should inspire empiricists to reconsider experimental designs for assessing how prey integrate multiple sources of information about predation risk (e.g., Caldwell et al. 2010) and provide theorists a foundation upon which to explore the interplay between density‐dependent predation, density‐dependent induction of antipredator responses, and their effects on population and community dynamics.

## Conflict of Interest

None declared.
